# Advantages and potential limitations of applying AFM kymograph analysis to pharmaceutically relevant membrane proteins in lipid bilayers

**DOI:** 10.1038/s41598-023-37910-7

**Published:** 2023-07-15

**Authors:** Katherine G. Schaefer, Arthur G. Roberts, Gavin M. King

**Affiliations:** 1 Department of Physics and Astronomy, University of Missouri, Columbia, MO 65211 USA; 2 Department of Pharmaceutical and Biomedical Sciences, University of Georgia, Athens, GA 30602 USA; 3 Joint With Department of Biochemistry, University of Missouri, Columbia, MO 65211 USA

**Keywords:** Membrane biophysics, Single-molecule biophysics, Nanoscale biophysics, Molecular conformation

## Abstract

Membrane proteins play critical roles in disease and in the disposition of many pharmaceuticals. A prime example is P-glycoprotein (Pgp) which moves a diverse range of drugs across membranes and out of the cell before a therapeutic payload can be delivered. Conventional structural biology methods have provided a valuable framework for comprehending the complex conformational changes underlying Pgp function, which also includes ATPase activity, but the lack of real-time information hinders understanding. Atomic force microscopy (AFM) is a single-molecule technique that is well-suited for studying active membrane proteins in bilayers and is poised to advance the field beyond static snapshots. After verifying Pgp activity in surface-support bilayers, we used kymograph analysis in conjunction with AFM imaging and simulations to study structural transitions at the 100 ms timescale. Though kymographs are frequently employed to boost temporal resolution, the limitations of the method have not been well characterized, especially for sparse non-crystalline distributions of pharmaceutically relevant membrane proteins like Pgp. Common experimental challenges are analyzed, including protein orientation, instrument noise, and drift. Surprisingly, a lateral drift of 75% of the protein dimension leads to only a 12% probability of erroneous state transition detection; average dwell time error achieves a maximum value of 6%. Rotational drift of proteins like Pgp, with azimuthally-dependent maximum heights, can lead to artifactual transitions. Torsional constraints can alleviate this potential pitfall. Confidence in detected transitions can be increased by adding conformation-altering ligands such as non-hydrolysable analogs. Overall, the data indicate that AFM kymographs are a viable method to access conformational dynamics for Pgp, but generalizations of the method should be made with caution.

## Introduction

Proteins that reside within the lipid bilayer membrane of a cell perform fundamental tasks. Biochemical activities carried out by membrane proteins include the transfer of information across the bilayer, often via highly specific signaling pathways, and the uphill transportation of molecules against gradients, frequently by harvesting the energy of ATP binding and hydrolysis^1^. This large family of proteins comprises approximately 25% of the proteome in any organism. Additionally, membrane proteins represent about 60% of all human drug targets and determine the ultimate disposition of many pharmaceutical agents^2–5^. Despite their pervasiveness in nature and pharmacological significance, a robust methodology for detecting time-dependent conformational changes which dictate membrane protein activity remains challenging.

Techniques that reveal protein conformations with the high scrutiny of atomic precision provide the foundation of modern structural biology. X-ray crystallography and, increasingly, cryogenic electron microscopy (cryo-EM) are the mainstays of this field. Though these methods have supplied critical knowledge for many membrane protein systems, they do not provide real-time data. Hence, kinetic information is lacking. Moreover, removal of the polypeptide chain from the bilayer may be required, which can affect protein conformation and function^6–8^.

The atomic force microscope (AFM) is a single molecule technique poised to advance high-precision studies beyond static snapshots into a regime of dynamic structural biology^9–12^. The AFM is well suited for studying membrane proteins in lipid bilayer environments. The sharp tip of the microscope probes membrane-external protein protrusions in fluid, in real space, and in real-time. The biochemical activity of membrane protein samples prepared for AFM study can be verified^13,14^. Though the lateral resolution of AFM on proteins is lower than the mainstay methods in the field, the vertical resolution is similar to X-ray crystallography (≤ 2 Å)^15^. Additionally, individual AFM images exhibit high signal-to-noise ratios that do not generally require averaging to interpret, in contrast to cryo-EM data^16^.

Many structural dynamic applications of AFM have involved proteins, such as bacteriorhodopsin, which form high-density two-dimensional arrays in the lipid bilayer and exhibit small membrane-external protrusions^17,18^. Membrane proteins with large azimuthally-asymmetric membrane-external domains, such as the P-glycoprotein (Pgp) transporter, which plays a critical role in cancer drug efflux^19^, have proven more challenging to study. Pgp is a member of the ubiquitous ATP Binding Cassette (ABC) transporter superfamily. This large protein pumps a chemically and structurally diverse range of small molecules across the cytoplasmic membrane, including many pharmaceuticals^20–22^. The Pgp transporter plays a major role in human cancer resistance because it pumps anti-cancer drugs from cancerous tumors before they can deliver their therapeutic payload^23^. Despite intense interest and many increasingly high-resolution structures, significant questions remain regarding the structural changes that underlie Pgp activity.

In this work we show structural transitions of Pgp in supported lipid bilayers at < 1 s timescales. AFM kymograph analysis is used in conjunction with imaging and simulations. Formed by disabling the slow axis of a raster pattern, kymographs comprise a set of one-dimensional time-dependent scans of membrane-external protein topography. Kymographs and other reduced dimensionality probing methods such as single point height versus time traces are increasingly being used in the AFM community to enhance temporal resolution^24–27^. Still, the advantages and limitations of these types of methods have not been well characterized, especially for sparse (non-crystalline) distributions of large pharmaceutically relevant membrane proteins such as Pgp. We address the issues of protein orientation, drift, and instrument noise as they represent potential roadblocks for identifying meaningful time-dependent conformational transitions. Additionally, kymograph analysis relies on the user’s ability to accurately begin acquisition on a suitable part of the protein to detect time-dependent conformational transitions. But what scanning angles and lateral positions yield the most useful results? What artifacts may be present in the data? Simulations were carried out to address these questions. We find that deceptively simple geometric changes in protein conformations or orientations translate into complex behavior. Experiments corroborated the theoretical analyses, showing an expected reduction in Pgp conformational dynamics upon addition of a non-hydrolysable ATP analog.

## Results

**Figure 1:**
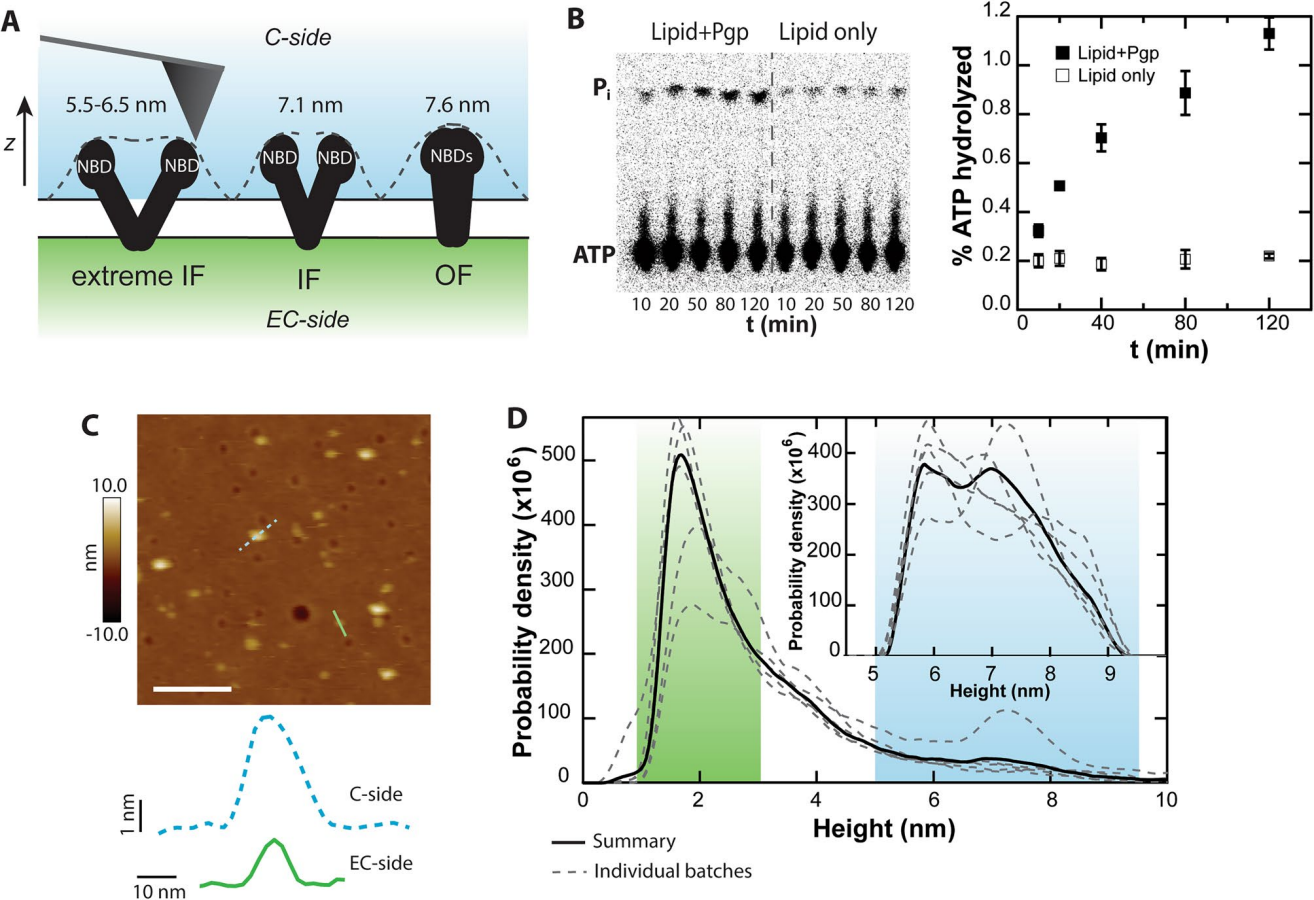
AFM imaging of active Pgp. (**A**) A cartoon shows the extreme IF, IF, and OF states of Pgp, as well as the expected heights of the C sides. The convolution of the tip with the protein protrusions is indicated (dashed line). Heights of the particles are measured in the *z*-dimension, normal to the bilayer. (**B**) A thin layer chromatography radiograph shows ATP activity sampled from glass-supported Pgp over the course of 2 h (left). A control assay is performed with bare lipid to compare to proteoliposomes reconstituted with Pgp. The right panel quantifies spot intensity to show percent ATP hydrolyzed by Pgp on a surface over time. (**C**) An AFM image of Pgp in a lipid bilayer on a glass surface is shown. Line scans of a larger C-side feature (blue dashed line) and a smaller EC-side feature (green solid line) demonstrate differentiation of sidedness in an image; scale bar = 100 nm. (**D**) Heights of all features are plotted using kernel density estimation to produce a smoothed histogram. Plotting multiple histograms of independent Pgp experiments shows some variation (dashed gray lines). The summary of all features is displayed as a solid black line (*N* = 92,212). Regions of the histogram corresponding to EC-side and C-side features are highlighted in green and blue respectively. C-side features are isolated for further analysis using a height window of 5.5 to 9 nm (inset; *N* = 9272).

Pgp conformational states are typically described as inward-facing (IF), in which the nucleotide binding domains (NBDs) are separated and the drug-binding sites are exposed to the cytosol, or outward-facing (OF), in which the NBDs are closely associated and the transport channel is open to the extracellular space. A cartoon displays Pgp in the IF and OF conformations (Fig. 1A). A range of IF conformations are possible, depending on the degree of NBD association, and an extreme IF conformation is also shown in the cartoon^28–30^. Note that both the IF and extreme IF states exhibit significant azimuthal asymmetry about the z-axis, which is normal to the bilayer. In the absence of torsional constraints, cylindrical asymmetry can complicate reduced dimensional scanning methods such as kymographs. In order to study Pgp in these conformational states, reconstituted Pgp was visualized in surface-supported bilayers via AFM. Cleaned glass coverslips were employed as supporting surfaces^31^. Pgp activity was verified with surface-supported samples prepared for AFM and assayed for inorganic phosphate release via thin-layer chromatography over two hours (Fig. 1B). The data show the hallmark of activity, enhanced hydrolysis only when the ATPase Pgp is present. All imaging and kymograph studies were kept within this two-hour time limit to ensure Pgp remained active for the duration.

A first task after AFM imaging is to differentiate the extracellular (EC) side from the cytosolic (C) side as reconstitution can scramble protein orientation. Due to the asymmetric structure of Pgp across the bilayer, geometric properties may be exploited to distinguish the smaller, extracellular loops from the larger protrusions caused by the cytosolic NBDs. The approximate heights for C-side features are given in Fig. 1A. EC-side features are 1 to 3 nm. Upon visualization of Pgp in the apo condition (no ligands added), AFM images were populated by punctate features with heights ranging from 1 to 9 nm (Fig. 1C, D). There are two distinct populations that are visible to the eye, one small and one large, corresponding with the EC side and the C side respectively. Figure 1C highlights two such features. The spatial resolution of AFM favors the z-dimension, or height, over the lateral dimensions, so the height metric is the focus of our studies. The heights of these features (about 2 nm and 7 nm, respectively) are in agreement with simulations of AFM images created by convolving a mathematical estimate of the AFM tip with coordinates of an apo Pgp structure (Protein Data Bank (PDB) code: 7OTI^32^). Following previous work, the tip geometry was modeled as two overlapping spheres, the lower one having a radius of 4 nm and the upper with a radius of 8 nm^33^. The expected heights of apo Pgp are 7.1 nm for the C side protrusion and 2.5 nm for the EC side, where the membrane bilayer background is placed based on the Orientations of Proteins in Membranes database^34^. Previous experiments with C-side-specific antibodies support this categorization of EC side and C side features^35^. Images are analyzed using the Hessian blob algorithm to isolate individual features and objectively extract topographic data such as the maximum height of the protein protrusion above the bilayer. The resulting height histograms exhibit a characteristic distribution having a primary peak around ~ 2 nm, the height of the EC side, which preferentially orients towards the AFM tip. The C-side features have a broad shoulder in the height histogram, extending to 9 nm (Fig. 1D). Some heterogeneity is observed between distinct experiments (Fig. 1D, gray dashed lines), which were acquired using several different Pgp preparations, but the general trend in the height distributions were similar. A summary trace of all samples totaling *N* = 92,212 Pgp protrusions (Fig. 1D, solid line) was used for further analysis. Pgp NBDs are highly dynamic, even in the apo state^30,36,37^. The dynamics give rise to the wide range of observed heights for the C-side features. We extrapolated from the apo Pgp simulation and used a wide height window of 5.5 to 9 nm to isolate features consistent with C-side topography. Taller features (> 9 nm), which could be aggregates, were rare (0.4% of the total) and excluded from the analysis. This threshold allows us to closely examine the structural diversity of over 9,272 C-side protrusions in a near-native environment (Fig. 1D, inset). When probing for conformational changes, care must be taken to compare apo and + ligand states from the same area of the same samples.

**Figure 2:**
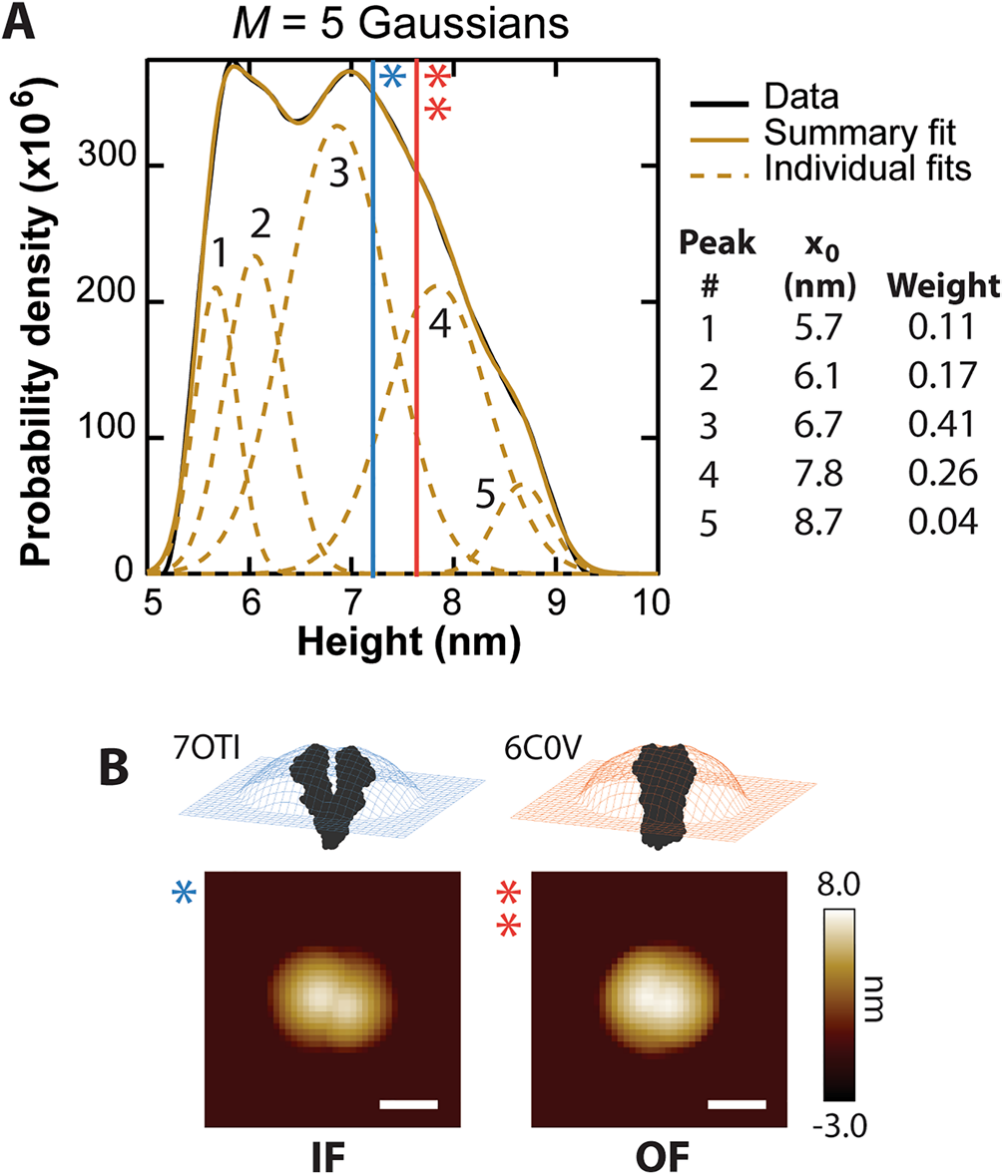
Analysis of C-side histograms. (**A**) Smoothed C-side height histograms are deconvolved by fitting with multiple Gaussians. Selection of an optimal number of model distributions is achieved by minimizing the BIC calculated for each additional Gaussian. For this summary histogram, *M* = 5 Gaussians were prescribed. The peak locations and weights of the individual Gaussians are shown. (**B**) Simulated AFM images of the IF conformation of mouse Pgp (PDB = 7OTI^32^) and the OF conformation of human Pgp (PDB = 6C0V^40^). The heights of these simulations are measured using Hessian blob analysis and plotted on the height histogram in panel (**A**) (blue = IF; orange = OF); scale bars = 10 nm.

The dynamics of the NBDs induce changes in C-side heights throughout AFM imaging. Within this complex energy landscape, the NBDs prefer certain conformations, notably the IF conformation, where they are separated, and the OF conformation, in which they are associated. The degree of NBD separation corresponds to certain height populations^28^. In order to deconvolve the C-side heights in an unbiased manner, we apply the Bayesian information criterion (BIC)^38^, which balances the goodness of fit with overfitting. After querying models with *M* = {1, 2, 3, 4, 5, 6} Gaussian distributions, a fit with the sum of 5 Gaussians was found to be optimal (Fig. 2A, Supplementary Information Section A, and Fig. S1). Populations were located at 5.7 ± 0.2, 6.1 ± 0.3, 6.9 ± 0.5, 7.8 ± 0.5, and 8.7 ± 0.3 nm (± σ). We note the location of the smallest height population, which also exhibited the least weight (11%, calculated as percent area of the fitted model), was sensitive to the lower cut off limit.

AFM simulations were performed on Pgp structures with the nucleotide-binding domains (NBDs) separated in the IF conformation and in the OF conformation. The apo state of Pgp has a wide dynamic range, but evidence suggests that the majority of apo Pgp resides in the IF conformation, in which the NBDs are separated^32,39^. The apo conformation is simulated as before, and the OF conformation is simulated using one of the few structures of this state (PDB: 6C0V^40^) where the two NBDs are in close proximity. The heights of the IF (*h* = 7.1 nm; Fig. 2B, left) and OF (*h* = 7.6 nm; Fig. 2B, right) conformations are plotted on the C-side height histogram as vertical lines to compare with experimental data (Fig. 2A).

Figure 2A shows that C-side heights range from 5.7 to 8.7 nm, consistent with Pgp shifting between the IF and OF conformations^32,39^. Of the individual protrusions we imaged, the largest percentage (~ 41%) exhibited a height of 6.9 ± 0.5 nm (Fig. 2A, peak 3). This population can be attributed to the IF state. It is within 2 Å of the expected height of the 7OTI structure in which the separation of NBDs causes a lower z measurement. A substantial fraction (~ 26%) of the protrusions were captured with taller heights 7.8 ± 0.5 nm (Fig. 2A, peak 4). This population is within 2 Å of the OF state, where the NBDs are associated with each other. Thus, 2/3^rds^ of the C-side protrusion population fall within 1 σ of the prominent previously characterized inward-facing or outward-facing states. Two smaller C-side protrusion populations had lower average heights of 5.7 and 6.1 nm, suggesting greater NBD separation and broader flexibility than in X-ray crystal and cryo-EM structures. This is evidence of extreme IF conformations with NBDs widely separated. A small C-side protrusion population (4%) had an average height of 8.7 nm. The 4 Å increase in height may be due to the vertical translation of Pgp in the membrane or the reorientation of the NBDs. Overall, these results indicate that apo-Pgp is predominantly found in inward-facing conformations.

Improved time resolution (~ 100 ms in our studies) may be achieved by disabling the slow scan axis of the raster pattern and making a stack of single-line scans in time. The resulting kymographs are widely used in the AFM community, but the advantages and potential pitfalls of the method have not been extensively probed, especially with regard to non-crystalline membrane proteins. Kymographs are subject to instrument noise and drift, reducing the accuracy of conformational transition detection. Additionally, the analysis method relies on the user’s ability to initiate the kymograph on the ideal part of the protrusion to detect transitions. Finally, both lateral and rotational protein diffusion or reorientation due to low protein density in the membranes can also affect data quality.

**Figure 3:**
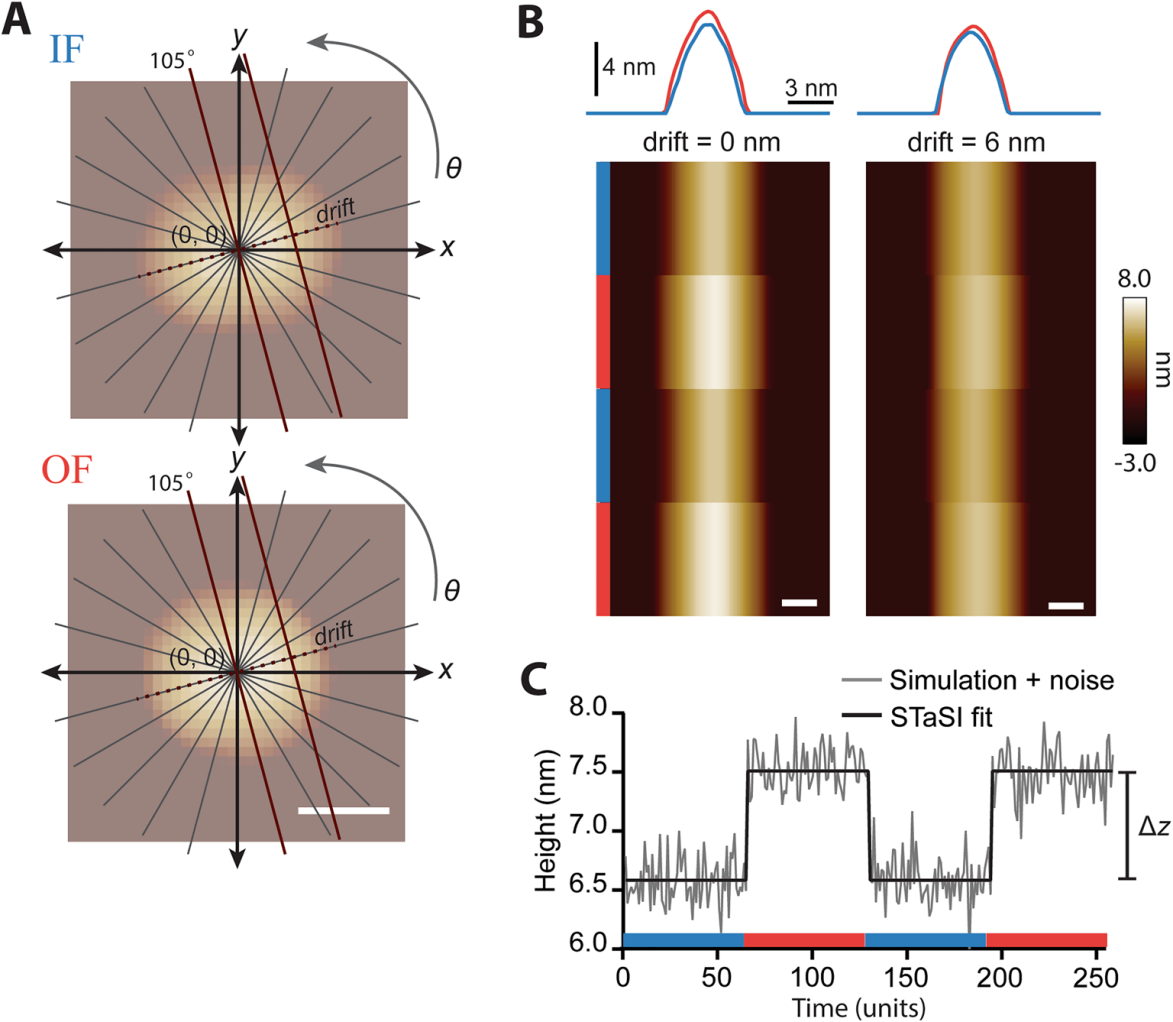
Kymograph simulations. (**A**) Kymographs are simulated from crystal structures by taking profile line scans across simulated AFM images. Thin gray lines represent the angles scanned. The dark red scan lines shown are at an angle of 105° with 0 nm and 6 nm displacement from the center of mass of each image. Two states are used: the IF conformation (blue) and the OF conformation (orange); the 10 nm scale bar applies to both images. (**B**) Line scans of the IF and OF states are displayed above each kymograph simulated at 105°. The left panel displays a kymograph at the center of mass (drift = 0 nm), and the right panel displays a kymograph with a drift of 6 nm. Line scans for each state are stacked for 64 time units each and alternated twice; this results in a kymograph with a 256 time unit duration exhibiting 2 states and 3 transitions. (**C**) The maximum height pixels are extracted from the kymograph simulated at 105° and 0 nm. AFM-typical Gaussian noise (σ = 2 Å) is added. The STaSI algorithm fits the simulated data with states and identifies transition points. The vertical height difference between states, ∆z, is shown to the right of the graph.

To explore the optimal conditions for kymograph acquisition, we simulated kymographs of Pgp C-side features in the IF and OF conformational states and determined how the scan angle and drift affect the accuracy of state detection. AFM kymograph simulations were produced by taking individual line scans across an AFM image simulated from the relevant crystal structure and stacking them in “time” (Fig. 3A,B). Each state repeats twice after a dwell time of 64-time units for a kymograph that lasts 256-time units. Thus we artificially impose that each kymograph has only two states and three transition points. We varied the scan angle from 0° to 165° in 15° steps, corresponding with the gray lines in Fig. 3A. Drift was simulated with 1 nm steps from the center of mass in both positive and negative directions. Kymographs were simulated until further steps in drift would cause the tip to lose contact with the protein. An example kymograph taken at the center of mass and a kymograph that has drifted 6 nm from the center of mass are shown in Fig. 3B. The amount of simulated drift varied depending on the scan angle, but ranged from 7 to 13 nm in either direction. For each kymograph simulation, the maximum heights were extracted and AFM noise was approximated as Gaussian with a standard deviation of 2 Å, characteristic of a commercial instrument under typical conditions (Fig. 3C, gray line). A total of 226 kymographs were simulated to cover the representative variation in scan angle and drift. A representative subset of simulations is provided in the supplement (Fig. S2).

Simulated kymographs were analyzed using the STaSI algorithm^24,41^. This algorithm was originally developed to analyze single-channel Fȍrster resonance energy transfer (FRET) data. It uses a Student’s T-test as a step detection method and fits the data with the optimal number of states. Notably, the STaSI algorithm does not require user input to determine state number. We adapted this method for our simulated kymographs and evaluated its accuracy at detecting transition points and enumerating states. Figure 3C shows the STaSI fit for a kymograph of the protein with a scan angle of 105° and no drift from the center of mass. At this particular orientation, the scan through the center of mass goes between the binding domains, giving rise to a line scan of IF Pgp 10 Å shorter than the line scan of the OF state. Change in the scan angle and drift yield varying maximum height values on the line scans, thus affecting the height difference (∆*z*) between states. For example, the 6 nm drift of the 105° scan yields ∆*z* = 4 Å, a reduction from the ∆*z* = 10 Å of the center of the mass scan. Depending on the signal-to-noise ratio (S/N), the addition of noise affects both state detection and transition point accuracy. For this discussion, S/N is defined as the ratio of the signal, ∆*z*, to the standard deviation of the Gaussian noise source, 2 Å. Most of our scans have a ∆*z* above the noise (S/N > 1), however, 11.5% of the simulations had a S/N < 1. Upon further examination, this subset of kymographs was generated with a lateral drift > 2 nm from the center of mass, meaning that kymographs beyond this point exhibit a non-zero likelihood of having ∆*z* below the noise. This leads to consequences for both the accuracy of state detection as well as the dwell time of each state.

**Figure 4:**
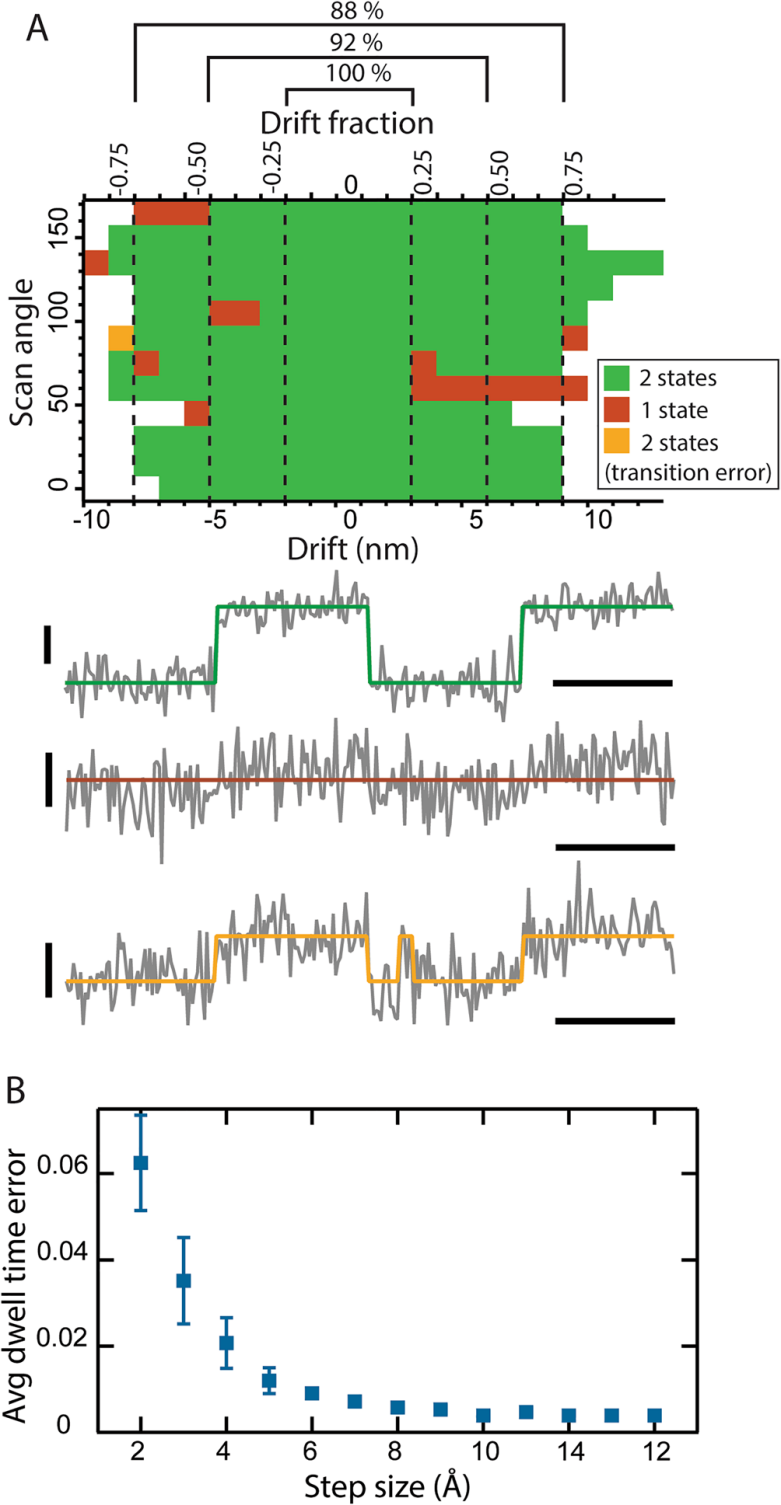
Accuracy of state and transition detection. (**A**) A graph displays the number of states detected for scan angle and drift from the center of mass (0 nm). An accurate state detection results in 2 states (green). There are 2 possibilities for inaccurate detection, either 1 state is detected (brown), or too many transitions are detected (gold). Examples of all three detection modes are shown below the graph; vertical scale bar = 5 nm, horizontal scale bar = 50 time units. Confidence measured as the percent correctly detected for each margin is shown above the plot. A unitless scale shows the fraction of the protein’s major axis. (**B**) Relative deviation from the expected dwell time (64 time units) is quantified for each detected state. Average errors for ∆*z* in 1 nm windows are calculated and plotted vs the transition step size (error bars = σ).

As drift increases, confidence in state detection decreases. Each simulated kymograph was designed to have only two possible states: IF or OF. In all cases within ± 2 nm from the center of mass, two states were accurately detected (Fig. 4A, green). A recurring error was observed with increasing frequency when the drift was allowed to progress to 3 nm and beyond; only one state was detected rather than two (Fig. 4A, brown). In one instance, the noise was mistakenly detected as a fleeting transition to one of the two states (Fig. 4A, gold).

To show the error in the transition point detection, deviation from the set dwell time of 64 units was calculated for the states in all kymographs. The average values for 1 nm vertical ∆*z* ranges are plotted to show that as ∆*z* approaches the noise level, the average dwell time error for a kymograph increases (Fig. 4B). The data reveal a maximum value of 6% for this error. Transitions for kymographs with step sizes below the noise were not detected.

**Figure 5:**
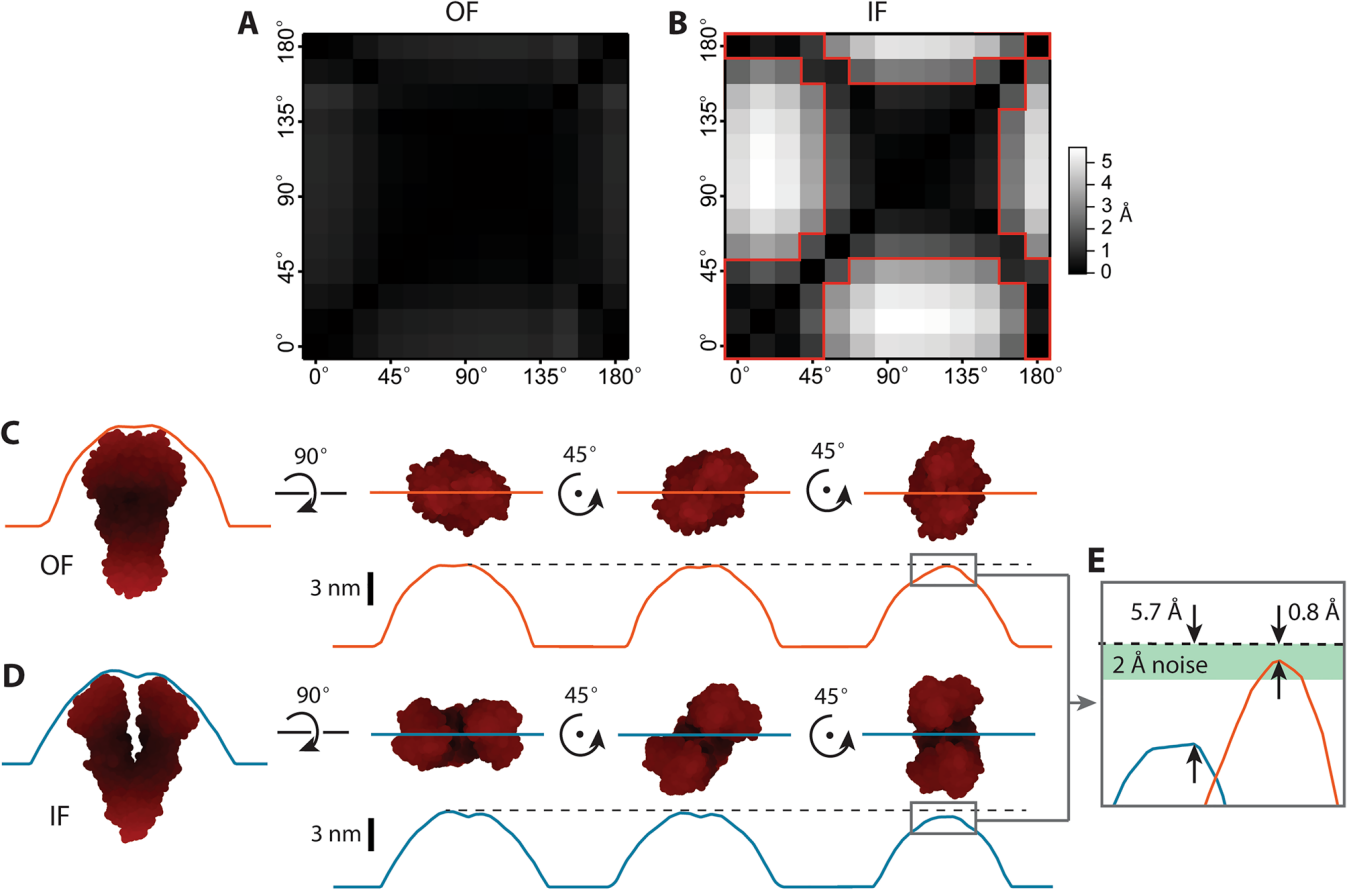
Rotational drift can produce artifactual transitions. To demonstrate the effect of rotational drift, height differences, ∆*z*, are calculated for rotations of the crystal structures for the OF (**A**) and IF (**B**) conformations. A matrix is built for the ∆*z* between a reference angle’s line scan and subsequent rotations. No rotations resulted in a detectable change for the OF conformation. In contrast, several in the IF conformation were above the noise (∆*z* > 2 Å), as indicated by the lighter regions outside the red boxes. (**C**, **D**) Cartoons demonstrate how different conformations yield different ∆*z* if rotations occur during a kymograph for OF & IF, respectively. Separation of the NBDs in the IF conformation gives rise to azimuthal-dependence in the measurement of maximum height, resulting in larger ∆*z* measurements. (**E**) The ∆*z* values are compared for the OF (orange) and IF (blue) states shown in (**C**, **D**), grey boxes. A green bar represents the noise level of 2 Å, the threshold for transition detection.

While acquiring kymographs, real-time corrections to the tip trajectory can be made to combat lateral drift. Conversely, for proteins outside of high-density 2D arrays which are also not torsionally constrained, it is not possible to deterministically control the orientation of a protein protrusion relative to the tip scan trajectory. Changes in this orientation while scanning, defined as rotational drift, may lead to detection of conformational state changes, even if there are none. Examination of height changes due to rotation shows that only certain angular displacements will result in a false transition detection due to a ∆*z* > 2 Å (Fig. 5). We tested the effect of rotational drift on the prominent Pgp conformations for scans through the centers of mass and found that rotation of the OF state does not yield any false positive transition detections (Fig. 5A), owing to this conformation’s high degree of azimuthal symmetry. The IF state, however, exhibits an azimuthally-dependent maximum height, leading to multiple possible angular displacements that result in artifactual transitions (Fig. 5B). The transitions with ∆*z* < 2 Å are shown as the darker areas of the matrix and outlined in red. Detectable transitions are the brighter areas. Some of these cases require a larger angular displacement, such as 0° to 90°, which has a ∆*z* = 5.7 Å. There are also a few instances which require a change of only 15° or 30°, particularly displacement from an orientation of 165° to 180° and vice versa. Closer examination of this displacement shows that 15° is the lower limit for artifactual transitions for Pgp, even when smaller angular displacements are evaluated (see Supplementary Information Section B and Fig. S3). Inspection of specific line scans show the IF conformation lends itself more to artifactual transitions with rotational drift due to its asymmetry (Fig. 5D) as compared to the OF conformation (Fig. 5C). Scans that go over the NBDs tend towards larger maximum heights than any scan that goes between the NBDs, leading to a Δ*z* above the noise (Fig. 5E). Additional control parameters, such as torsional constraints or ligand-induced structural transitions, can temper the deleterious effects of rotational drift.

**Figure 6:**
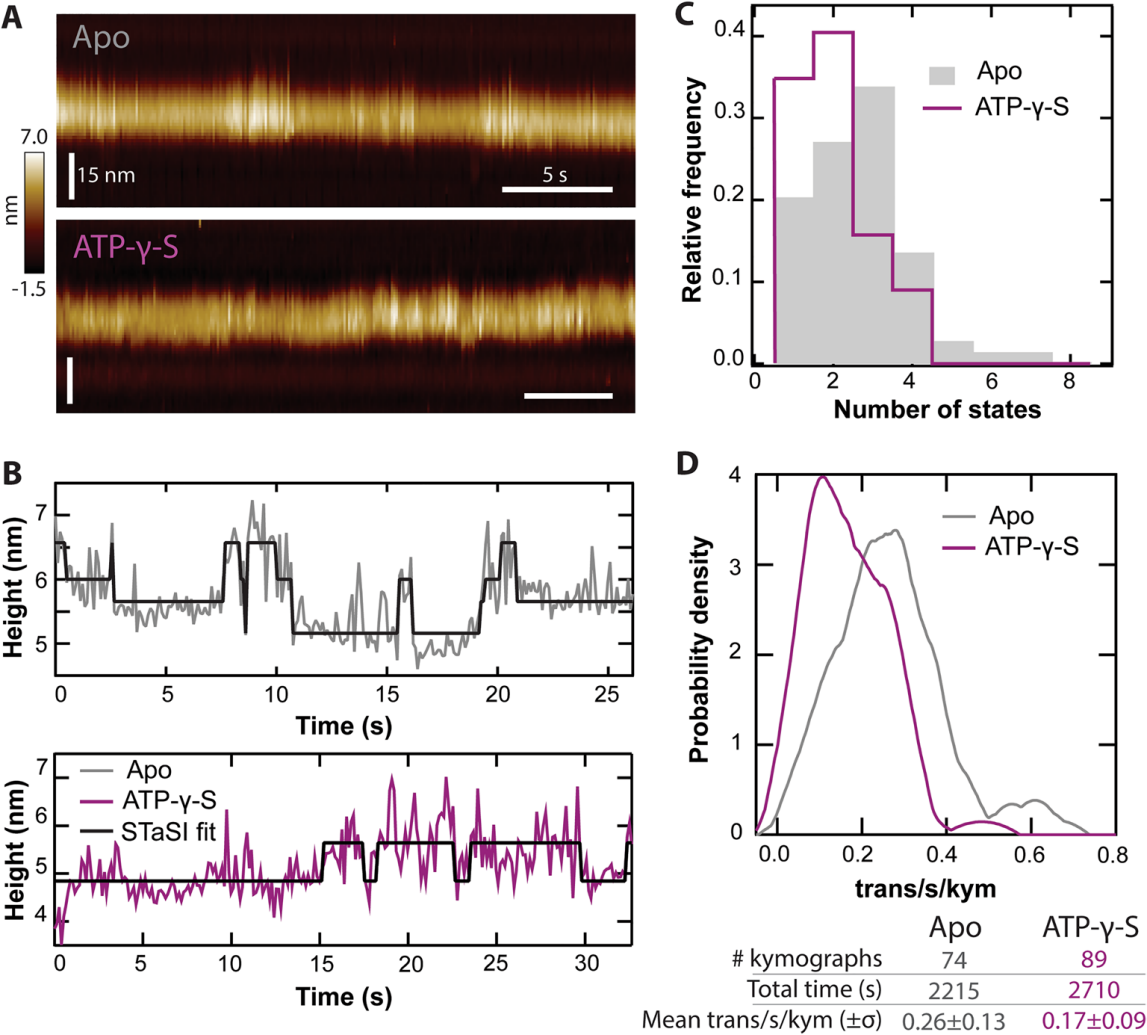
Pgp kymograph results. (**A**) Representative kymographs of C-side Pgp protrusions ± ATP-γ-S (Apo scan rate = 9.8 Hz; + ATP-γ-S scan rate = 7.8 Hz). (**B**) Kymograph maximums are extracted with custom software and analyzed with the STaSI algorithm. Here, 3 states are detected for apo Pgp (gray) and 2 states are detected for Pgp in the presence of ATP-γ-S (purple). (**C**) Histograms of the number of states detected for each kymograph show a peak at 3 states for the apo condition and at 2 states in + ATP-γ-S condition. (**D**) The number of transitions per second are calculated for each active kymograph. Smoothed histograms show a shift downwards in the trans/s/kym after the addition of ATP-γ-S. There is a ~ twofold reduction in the mean trans/s/kym from apo to + ATP-γ-S. The table summarizes statistical values for the kymograph data.

The dynamics of the NBDs are coupled with the ATP hydrolysis cycle^42–45^. Adding a non-hydrolysable analog such as ATP-γ-S arrests the cycle in an intermediate ATP-bound pre-hydrolysis state. To evaluate these dynamics in a single molecule AFM measurement, Pgp samples were imaged first in the apo state, then saturating (5 mM) ATP-γ-S was added. Representative kymographs for individual apo and + ATP-γ-S C-side features and the resulting STaSI fits are shown (Fig. 6A,B). The apo kymographs most frequently fluctuated between 3 different states, as opposed to + ATP-γ-S, which more frequently exhibited one or two states (Fig. 6C). Relative activity for mobile protrusions (those with more than one observed state) was quantified as the number of transitions detected per second per kymograph (trans/s/kym) (Fig. 6D). With the addition of ATP-γ-S, the activity transitioned from a mean value of 0.26 trans/s/kym to 0.17 trans/s/kym. This calculation was only performed on dynamic features defined as protrusions exhibiting ≥ 1 transition per kymograph. Kymographs showing fewer transitions were considered inactive Pgp. Of the 74 apo kymographs, 15 were inactive (20%), and of 89 + ATP-γ-S kymographs, 31 were inactive (35%).

**Figure 7:**
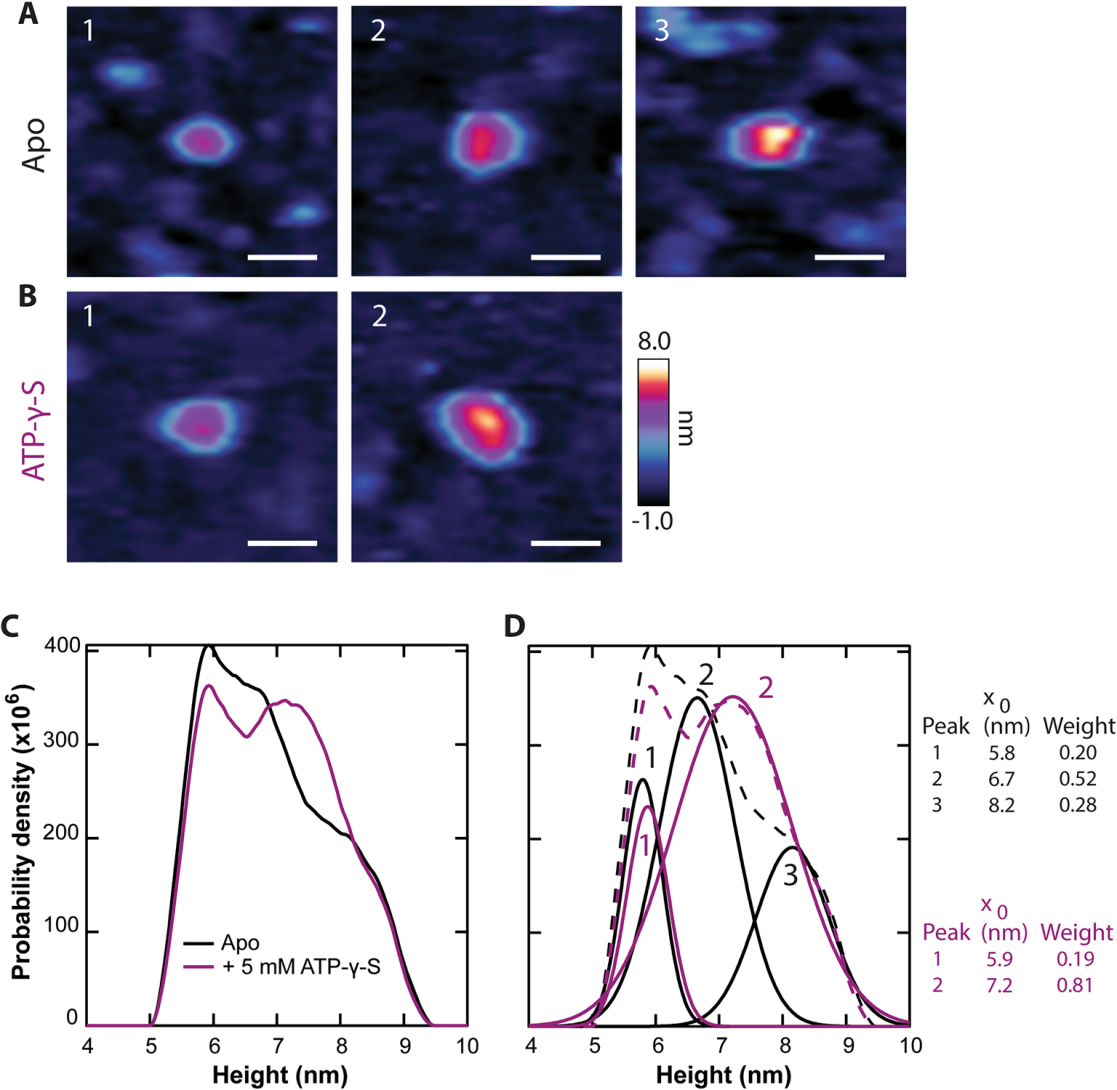
AFM imaging of Pgp + ATP-γ-S. Representative C-side features are displayed for the apo (**A**) and + ATP-γ-S (**B**) conditions whose heights correspond with peaks identified in panel (**D**); scale bars = 20 nm. (**C**) Height histograms compare the C-side Pgp in the absence (black) and presence (purple) of ATP-γ-S (N_apo_ = 929, N_ATP-γ-S_ = 900). (**D**) Both conditions are analyzed by deconvolving the histograms with the BIC method. The apo state requires *M* = 3 Gaussians and the + ATP-γ-S state requires *M* = 2. The resulting peak positions and weights are listed.

The reduction in conformational dynamics observed in kymographs was recapitulated in image data (Fig. 7). To more precisely compare C-side protrusions before and after the addition of a nucleotide, the same nominal areas of the same samples were studied. Here, the apo Pgp height histogram exhibited three populations, whereas the + ATP-γ-S condition had two populations (Fig. 7C,D). In the apo Pgp conformational state, there were two taller populations at 6.7 ± 0.6 nm and 8.2 ± 0.6 nm, which comprised 80% of the weight of the histogram. The addition of ATP-γ-S collapsed the two taller C-side populations into a single peak located at 7.2 ± 0.9 nm. Individual particles representative of each population are displayed for both the apo (Fig. 7A) and ATP-γ-S (Fig. 7B) conditions. The weight and location of the shortest height population were essentially invariant (within experimental uncertainty) upon ligand addition (apo: 5.8 nm, 20%; + ATP-γ-S: 5.9 nm, 19%). Note the reduction in BIC-determined apo states from 5 to 3 can be attributed to sampling a smaller number of C-side Pgp protrusions in these ± ATP-γ-S experiments (*N* = 929) compared to the summary distribution shown in Fig. 2A (*N* = 9,272). This approach reduces the effects of experiment-to-experiment variation at the expense of overall statistical weight.

## Discussion

The power of biological AFM is demonstrated in the case of Pgp, a pharmaceutically significant ABC transporter that exhibits complex conformational dynamics in the membrane. By starting with a small ensemble analysis of AFM images, snapshots of state distribution are obtained for apo Pgp. Cryo-EM structures provide insight into the relationship between membrane-external protrusion height and conformational state for the C-side features, however, the AFM measurements reveal a broader height range than the cryogenically preserved static structures. The C-side protrusions exhibited ≥ 2 topographical conformations, as isolated by the BIC, that are differentiable in height. Two prominent apo states appear very similar (within 2 Å) to cryo-EM structures of either the IF or the OF conformations. These two conformations comprise 67% of total. AFM measurements revealed ≥ 1 lightly populated shorter height states which likely correspond to the NBDs being more widely separated, which can be considered an extreme IF conformation^28^. Indeed, there may be more than one IF conformation according to other studies of both Pgp and related ABC transporters^29^. Researchers have shown that NBDs in the apo state fluctuate widely between IF and OF, though it is primarily agreed that the IF conformation dominates in the absence of substrate or nucleotide^29,36,37^. The dynamics of the IF state alone may allow more access to the binding pocket, thus a possible contribution to the high substrate promiscuity of Pgp and related transporters^36^.

In the OF state the NBDs are closely associated. It is thought that ATP hydrolysis is facilitated by this association. Studies suggest intermediates exist, such as post-hydrolysis ADP-bound^37,46^ and pre-hydrolysis ATP-bound states, notably a partial occluded state in which one of the two ATP binding sites is occupied and non-exchangeable^36,47^. For all of these, however, it is unclear how closely the NBDs are associated and whether the height difference would be distinguishable in AFM. Close association of the NBDs leads to a height increase as measured by AFM. We observe a higher-than-expected number of individual protrusions in an OF state for apo Pgp. Previous work in cryo-EM, single-molecule FRET, and hydrogen–deuterium exchange mass spectroscopy report low single-digit percentages of apo Pgp in the OF state^29,30,48^. Our AFM measurements in fluid lipid bilayers have 26% of C-side protrusions in a height range consistent with the OF state. Additionally, we identify a lightly populated (4% of total) “tightly-closed” OF population extending 8.7 nm above the lipid bilayer surface. To our knowledge, this state has not been previously reported.

We probed the structural implications of Pgp binding the non-hydrolysable ATP analog ATP-γ-S. This ligand traps Pgp in a pre-hydrolysis state, halting the cycle and perhaps arresting the motion of the NBDs^47,49^. Imaging indicated that the presence of ATP-γ-S reduces the number of Gaussians needed to fit the C-side height histogram compared to the apo state (Fig. 6). Furthermore, in saturating ATP-γ-S, C-side protrusions preferred certain conformations, intermediate between the two prominent apo states measured on the same samples. These results indicate reduced dynamics in the presence of the non-hydrolysable ATP analog and are consistent with the kymograph data. Interestingly, the height of the + ATP-γ-S state (7.2 ± 0.9 nm) is more characteristic of the apo condition than the OF conformational state. This indicates that the NBDs are not as closely associated as they could be when bound to this ATP analog. Others have also observed that Pgp is often seen in the IF conformation for ATP-γ-S^48^ and other analogs and ATP^29^.

Analysis of AFM kymographs usually consists of a transition-finding algorithm and quantification of dynamics. In addition, researchers have quantified errors with respect to the signal-to-noise ratio^24^. Here we emphasize the effect of protein protrusion geometry on kymograph analysis and explore limitations based on typical instrument performance. We simulated kymographs using cryo-EM structures of the IF and OF states and processed them through STaSI, a state-fitting algorithm. Protrusion geometry is most likely to affect the change in state height, ∆*z*, as the scan angle changes and the protein drifts with respect to the tip. For Pgp, deceptively simple geometric changes translate into complex behavior for ∆*z* (see Supplementary Information Section C and Fig. S4). Our kymograph simulations give insight into how the scan angle plays a role in degenerate ∆*z*’s for the same radial displacement from the center. In all cases, a higher ∆*z* and therefore, higher S/N is preferable for state detection. This analysis can be generalized to a number of scenarios, allowing an AFM operator to optimize kymograph measurements for transition detection, especially for systems that experience only subtle changes in height during state transitions. Lateral drift, in some cases, may increase the likelihood of state detection if there is some lateral change that increases ∆*z*. An example beyond the Pgp system would be SecA in the general secretory system of *Escherichia coli*, which is an ATPase that has a large mechanical motion of the precursor-binding domain. These conformations can be detected as a fluctuation in kymograph width^50^ or height if scanning in the location of this motion.

We further examined the effects of tip-sample drift, which remains a persistent issue in AFM instrumentation^51^. We find that while drift does have an appreciable effect on accurate state detection, it is not, perhaps, not as drastic as one may expect. A measurable error in transition state detection sets in after drifting > 2 nm away from the Pgp protrusion center. This amount of drift concurrently reduces the measured height of C-side Pgp protrusions to below ~ 5 nm. We note that this significant height change can act as a threshold to inform the user when the tip has drifted away from the optimal scanning pathway. Alternatively, the instrument drift rate can be used as a trigger for re-centering. Drift rates vary from instrument to instrument and also from day to day on the same instrument. A typical drift rate value is 2 nm/min even after an instrument is allowed to “settle”^51^. However, higher drift rates are frequently observed. The drift rate can be used to set an upper limit for kymograph duration (< 1 min for Pgp). That said, even when scanning on the edge of a Pgp C-side protrusion (drift > 5 nm away from the center), our analysis shows 88% transition detection confidence (Fig. 4A). In Fig. 6A, the apo kymograph experiences notable drift, estimated to be about 9.5 nm/min. The duration of the kymograph is 26.2 s, meaning the particle drifted ~ 4 nm relative to the tip. In this zone we identified that 92% of transitions are still detectable. However, this confidence metric is conservative, accounting for deleterious drift directionalities, which may not be present in this kymograph. Note that drift perpendicular to the kymograph scan direction can cause errors, whereas drift parallel to the scan direction is innocuous (see Fig. S5).

An additional consideration is the possibility of angular displacement (rotation) of the proteins in the bilayer. In the native membrane, many proteins are thought to undergo fast rotational diffusion^52,53^. Rotational diffusion coefficients depend on a number of factors, such as protein size and membrane environment^53,54^. Several experimental efforts have focused on the photoreceptor rhodopsin, resulting in diffusion coefficients which range from 0.25 × 10^5^ to 1.0 × 10^5^ rad^2^/s^55–57^. Theoretical models show the slowing effect of membrane crowding^54^. In AFM, the presence of a supporting surface is likely to introduce some resistance to rotational motion. Sullivan and colleagues showed that in the model case of carbon nanotubes in supported lipid bilayers, rotational motion could be observed with AFM. The carbon nanotubes’ average rotational step was 20°–50° s^−1^, but rotations as large as 180° s^−1^ were observed^58^. Carbon nanotube diameters are similar to that of a typical membrane protein (a few nm); their behavior may mimic that of proteins in a supported lipid bilayer. Yet, proteins are more topographically complex and exhibit many distinct chemical moieties at their periphery. These attributes are likely to provide torsional constraints via substrate interactions that are not present in carbon nanotubes and indeed appear to be present for Pgp (see Supplementary Information Section B, and Figs. S6, S7). If torsional constraints are absent, our simulations show that rotations of Pgp can give rise to erroneous detection of transitions, even if the protein conformation remains unchanged (Fig. 5). When studying protein protrusions that yield AFM topography with azimuthally-dependent maximum height, it may be difficult to say with confidence whether detected transitions result from conformational changes or rotations. As demonstrated here, this uncertainty can be minimized by employing an additional control parameter, in particular, altering the Pgp conformational state via addition of ATP-γ-S. Additionally, orientational analysis of a single Pgp protrusion shows ~ 1° rotational deviation occurring within the timescale of a single kymograph (Fig. S7), well below the ~ 15° threshold for erroneous transition detection (see Fig. S3).

Kinetic information, such as conformational transition rates, can be garnered through AFM kymograph analysis. We evaluated how common experimental challenges propagate into time domain calculations. We found that the average dwell time error is minimally affected by lateral drift. Even when operating at the limit of state transition detection, the maximum dwell time error only reached 6% (Fig. 4B). Under these idealized conditions kinetics calculations are susceptible to very small errors.

Kymographs of Pgp in the presence and absence of ATP-γ-S supported these conclusions. The addition of the non-hydrolysable analog reduced the measured Pgp dynamics on the 100 ms timescale. AFM imaging can only give details of the current conformational states as snapshots, whereas kymographs give information with higher time resolution about individual protein protrusions. Mirroring the reduction of Pgp populations in the image data, the kymographs also exhibited a reduction in the number of states detected in individual protrusions when ATP-γ-S was added. Recapitulation of the expected ligand-induced change is consistent with the notion that rotational drift artifacts are negligible in the Pgp kymographs. We posit that this is due to torsional constraints provided by non-specific binding between the surface-proximal protein domain and the glass support. We also learned from the kymographs that this state reduction was accompanied by decreased activity in the time domain, as evidenced by fewer conformational transitions recorded per second. In summary, we demonstrated the strengths and discussed some limitations of AFM kymograph analysis using Pgp as a pharmaceutically-relevant membrane-embedded model transporter. AFM scanning with reduced dimensionality is a powerful technique for studying active biological macromolecules, and careful consideration of how this technique is applied can further enhance the information content of the data it provides.

## Methods

### Sample preparation

The his-tagged wild-type mouse Pgp (Abcb1a, MDR3) was purified from *Pichia* (*P.*) *pastoris* using nickel–nitrilotriacetic acid (Ni–NTA) (Thermo Fisher Scientific) followed by diethylaminoethyl cellulose (DEAE) resin (Thermo Fisher Scientific)^59,60^. Proteoliposomes were prepared with a lipid-to-protein ratio of 6.25 µM Pgp (mg ml^−1^ lipid)^−1^ using 80% wt/vol *E. coli* Total Lipid Extract (Avanti Polar Lipids) and 20% wt/vol cholesterol^61–63^. First, a thin lipid film was created by evaporating the mixture of lipid extract and cholesterol dissolved in chloroform. The dried lipid film was rehydrated with 0.1 mM triethylene glycol diamine tetraacetic acid (EGTA) and 50 mM Tris–HCl (pH 7.4) and followed by ten freeze–thaw cycles in liquid nitrogen, generating liposomal vesicles of various sizes. The liposomes were extruded through a 400 nm Millipore filter by a LIPEX extruder (Evonik, Essen, Germany), which resulted in an average liposome size of 250 nm. Pgp solubilized by DDM was reconstituted into 250 nm liposomes using a previously described procedure so that the majority of the nucleotide-binding domains (NBDs) projected outside^63^. Proteoliposomes were stored in 4-(2-hydroxyethyl)-1-piperazineethanesulfonic acid (HEPES) buffer (20 mM HEPES, 100 mM NaCl, 5 mM MgCl_2_, 2 mM DTT, pH 7.4)^64^. The concentration of protein reconstituted in liposome was determined with the DC Protein Assay Kit II (Bio-Rad, Hercules, CA) or using the 280 nm Pgp absorption extinction coefficient of 1.28 mg^−1^ ml^−1^ (0.181 μM^−1^ cm^−1^)^59^. The average hydrodynamic diameter of the reconstituted proteoliposomes determined by dynamic light scattering (DLS) was 250 nm, as noted previously^28^.

### Atomic force microscopy

Glass coverslips were prepared by KOH etching^31^. Immediately before experiments, coverslips were quartered and plasma cleaned, then affixed to Teflon-covered metal disks with 5-min epoxy. Aliquots of proteoliposome stock were removed from – 80 °C and thawed. The sample was diluted in imaging buffer (20 mM HEPES, 100 mM NaCl, 5 mM MgCl_2_, pH 7.3) to 100 nM Pgp. Then 100 μL was added to the prepared glass surface. Samples were incubated at room temperature for 30 min to allow proteoliposomes to rupture and spread on the surface. Excess proteoliposomes were removed by rinsing 5–6 times via buffer exchange (100 μL volumes exchanged with a pipette). Images were collected in tapping mode (Cypher, Asylum Research) with biolever mini tips (Bruker, k ~ 0.1 N/m, f_o_ ~ 25 kHz in fluid) and tip-sample forces maintained below 100 pN. After AFM imaging apo-Pgp, 5 mM ATP-γ-S (Millipore-Sigma) was added to the sample and incubated in the AFM for 10 min (*T* ~ 35 °C). Samples were not moved during this process, so images collected are nominally in the same area for the + ATP-γ-S condition. Images were collecting using a scan rate of ~ 3.0 Hz (frame rate ~ 0.35 min^−1^) and kymographs were collected with scan rates of 6.5 to 9.8 Hz. All images are processed with second-order flattening using built-in software (Asylum Research). Custom software was used for streak removal and feature detection^65^ and smoothed histograms were generated via Epanechnikov kernel density estimation^66^. C-side features were isolated from data sets by filtering out features with heights lower than 5.5 nm and higher than 9 nm. BIC analysis was accomplished in Igor 7 using a custom algorithm (see Supporting Information Section A). When fitting the models, all parameters in Gaussian sums were allowed to vary.

### Simulation of AFM images and kymographs

AFM images were simulated from the crystal structures 7OTI for the IF state and 6C0V for the OF state. Membrane placement was determined using the OPM database, with 4.8 Å added to account for the lipid head groups. The tip geometry was deduced from experimental data to be two overlapping spheres, the apex sphere having a 40 Å radius and the base sphere having an 80 Å radius^33^. The images had a lateral resolution of 10 Å per pixel. Kymographs were simulated from these images by extracting line scans from the 2D plots. These scans were collected for 0° to 165° in 15° increments. For each angle, drift from the center of mass was also simulated in 1 nm steps until the model tip reached the edge of the feature. Line scans for the IF and OF states were each stacked in “time”, alternating twice with a dwell time of 64 time units for each state. The final result was a kymograph with two states and three transitions, lasting 256 time units. To generate single-dimensional traces of the kymograph backbone, the maximum pixels were extracted and Gaussian noise added. Gaussian noise was generated using a Box-Muller algorithm and had a standard deviation of 2 Å.

### Surface-absorbed activity assay

Samples were prepared for AFM as described above. After rinsing, 100 μL 3 mM radiolabeled [γ-^32^P] ATP (0.908 nCi/μL, PerkinElmer) in imaging buffer was added to the surface. At set time points, 4 μL of the surface solution was sampled and mixed with 4 µL of 50 mM ethylenediaminetetraacetic acid (EDTA). The resulting solutions for each timepoint were spotted onto a thin-layer chromatography (TLC) plate (Millipore Corporation) and allowed to dry. Separation of species was performed by submerging the bottom of the TLC plate in 125 mM KH_2_PO_4_ solution for 1 h. Then the plate was dried and exposed to a phosphor imaging plate (Fujifilm BAS-MS 2340) for 15 min in a closed cassette. The imaging plate was scanned in a phosphor imager (FLA3000) and analyzed using ImageQuant software. The percent ATP hydrolyzed was quantified as the relative amount of radioactive inorganic phosphate species that migrated from the ATP spot.

## Supplementary Information

Supplementary Information.

## Data Availability

The datasets generated during and/or analyzed during the current study are available from the corresponding author on reasonable request.
