# Supplementary material for: Advantages and potential limitations of applying AFM kymograph analysis to pharmaceutically relevant membrane proteins in lipid bilayers
Source: Sci Rep. 2023 Jul 15;13:11427. doi: 10.1038/s41598-023-37910-7 (PMC10349840; doi:10.1038/s41598-023-37910-7)
Supplement: Supplementary file 1 — Supplementary Information. [file 41598_2023_37910_MOESM1_ESM.pdf]

Supplementary Information

Advantages and potential limitations of applying AFM kymograph analysis to pharmaceutically relevant membrane proteins in lipid bilayers

Katherine G. Schaefer[1], Arthur G. Roberts[2,*], Gavin M. King[1,3,*]

[1]Department of Physics and Astronomy, University of Missouri, Columbia, MO 65211

[2]Department of Pharmaceutical and Biomedical Sciences, University of Georgia, Athens, GA 30602

[3]Joint with Department of Biochemistry, University of Missouri, Columbia, MO 65211

Contents

Section A: Bayesian information criterion

Section B: Reduced drift from particle pinning

Section C: Generation and interpretation of Δz vs lateral drift heat map

Figure S1: Bayesian information criterion plots and parameters

Figure S2: Kymograph simulation examples

Figure S3: Resolved artifactual transitions

Figure S4: The effect of lateral drift on Δz

Figure S5: Drift directionality

Figure S6: Lateral drift

Figure S7: Rotational drift

§A: Bayesian information criterion. The Bayesian information criterion (BIC) was used to objectively select a model for P-glycoprotein (Pgp) data. The BIC is defined as

$$BIC = -2\ln(\mathcal{L}) + k\ln(N)$$

where $\mathcal{L}$ is the maximized likelihood function for the model being tested, k is the number of free parameters in the model, and N is the size of the data set [1]. In our algorithm, we maximized the log-likelihood function, as it is equivalent to maximizing the likelihood function. The BIC determines which model is a good fit while penalizing each additional free parameter. After calculating the BIC for each model, the one with the minimum BIC is determined to be optimal for describing the data. In this way, we select the best model from a chosen set with little bias. For simplicity, we chose to test a set of models in which each model was a sum of M normal distributions, with M ranging from 1 to 6 (**Fig. S1A**). In addition to calculating the BIC, we also calculated the posterior model probability, p_i, for each model, indexed i (**Fig. S1B**) [2].

$$p_i = \frac{\exp(-\Delta_i/2)}{\sum_{r=1}^{M}\exp(-\Delta_r/2)}$$

Here, Δ_i is the difference between the individual model BIC and the best BIC. This method was applied to Pgp distributions in order to describe the number of conformations the protein sampled during imaging.

§B: Reduced drift from particle pinning. To investigate whether lateral drift is due to instrumental drift or due to Pgp diffusing in the bilayer, we took multiple image stacks and observed displacement of features over time. An example of images from one of these stacks is displayed in **Figure S6A**. Conveniently, the glass surface has naturally occurring pores (voids) which serve as fiducial marks for this measurement. We tracked Pgp features relative to these fiducial marks to determine whether drift was instrument or diffusion-driven. From three separate

stacks, we found the displacement between a protein protrusion and fiducial mark to be consistent, with standard deviations ranging from 4-7 Å over several minutes (**Fig. S6B**). This indicates that the lateral motion of observed features is likely due to instrumental drift. The registered drift with fiducial marks is also evidence of protein pinning to the supporting surface, reducing the protein's ability to diffuse freely. Additional agreement analysis is performed for a series of C-side protrusions, comparing each to a simulated image (PDB: 7OTI [3]) (**Fig. S7A** and **B**) [4]. The output of the orientation displays minimal change over time, with the mean angle being 174° ± 6°. The angular drift rate of the particle (ω) is 0.04° ± 0.05° s^{-1}, determined by fitting a line to the data. For the timescale of a kymographs, about 25 s, this implies an angular displacement of only 1° throughout the kymograph period. According to analysis of rotational drift and the possibility of false transition detections, this is not sufficient to produce an error. Closer examination of artifactual transitions reveals that 15° is the lower limit, even when finer angular displacements are evaluated (**Fig. S3A** and **B**). Reduced rotational drift is expected if a particle has at least two points of contact with the glass surface. This is consistent with previous neutron scattering measurements which predict the space between the glass and the bilayer is ~1 nm [5]. For a C-side feature exposed to the AFM tip, the EC-side would be confined in this space and would likely contact the surface, as the EC loops protrude 2-3 nm from the lower leaflet.

§C: Generation and interpretation of Δ*z* vs lateral drift heat map. We sought to quantify conformational height changes, Δ*z*, in a meaningful way. A heat map of Δ*z* versus drift was generated for all scan angles (**Fig. S4A**). Despite the relative simplicity of the two-state (open-closed) simulation, the data demonstrate a rich texture in the heat map. To aid interpretation, we further simplified the simulation from PDB coordinates to basic three-dimensional shapes. We

used a single sphere of radius r = 3.8 nm to approximate the closed state as shown in **Figure S4B**, then geometries of increasing complexity as the open state: an oblate spheroid (a = 3.9 nm, b = 3.55 nm), an ellipsoid (a = 3.09 nm, b = 5 nm, c = 3.55 nm), and two overlapping spheres (r = 3.55 nm, a = 2 nm), representing the increased separation of the NBDs in the open state. Because of the radial symmetry of the oblate spheroid, the resulting heat map is simple. Every scan angle produces the same drift-dependent Δz, which decreases from a maximum at the center of mass (*). The drift is demonstrated in **Figure S4C**, where two scan angles, 0° and 90°, with the same drift produce the same Δz. However, the ellipsoid is narrow on one axis and wide on another, imposing two trends in drift-dependent Δz as the kymographs sweep through the different scan angles. For the set of angles normal to the major or minor axes of the ellipse, Δz increases with drift (**), whereas for the intermediate angles, Δz decreases (**Fig. S4B**). Again, this is evident for two scan lines with similar drift but different angles (**Fig. S4C**). This trend can be observed in the main simulation (**Fig. S4A**), indicating a similar change in geometries for the PDB structures. Finally, an overlapping sphere geometry gives insight into the spread in Δz at the zero drift position. The kymograph analysis software compares the maximum pixels in the measurement. For the overlapping sphere, a scan between the spheres will have a lower maximum height than a scan across the tops of the two spheres (**Fig. S4C**). Similarly, separated NBDs in the inward-facing state of Pgp will produce a different Δz at the center of mass depending on the scan angle (***). With a better understanding of the complexities in the heat map, we can see that most scans have a Δz above the noise (S/N > 1). Based on our heat map, kymographs that drift >2 nm away from the center exhibit a non-zero likelihood of having Δz below the noise. This results in consequences for both the accuracy of state detection, and the dwell time of each state.

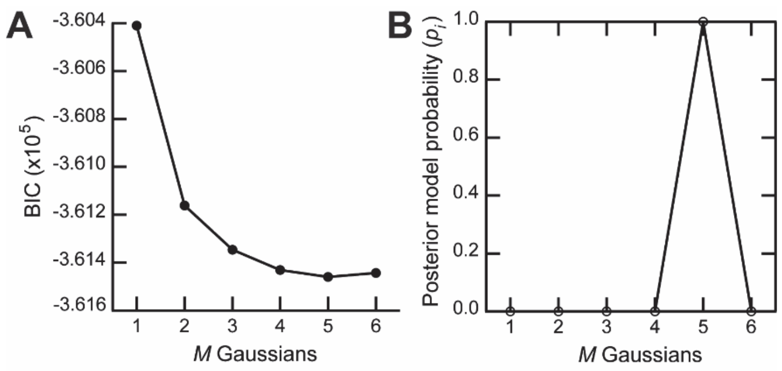

C

M Gaussians	*k*	BIC	Δ_i	p_i
1	3	-360,410	1048.91	0
2	6	-361,161	298.969	0
3	9	-361,347	113.531	2.223×10^{-25}
4	12	-361,430	29.8125	3.359×10^{-7}
5	15	-361,460	0	0.9998
6	18	-361,443	17.4062	1.660×10^{-4}

Figure S1. BIC plots and parameters. (A) The BIC is calculated for each model of increasing Gaussian distributions and plotted to show the occurrence of the minimum at *M* = 5 Gaussians. **(B)** Posterior model probability is calculated for each model, showing a high probability of *M* = 5 Gaussians being the correct model in the set. **(C)** Table of parameters.

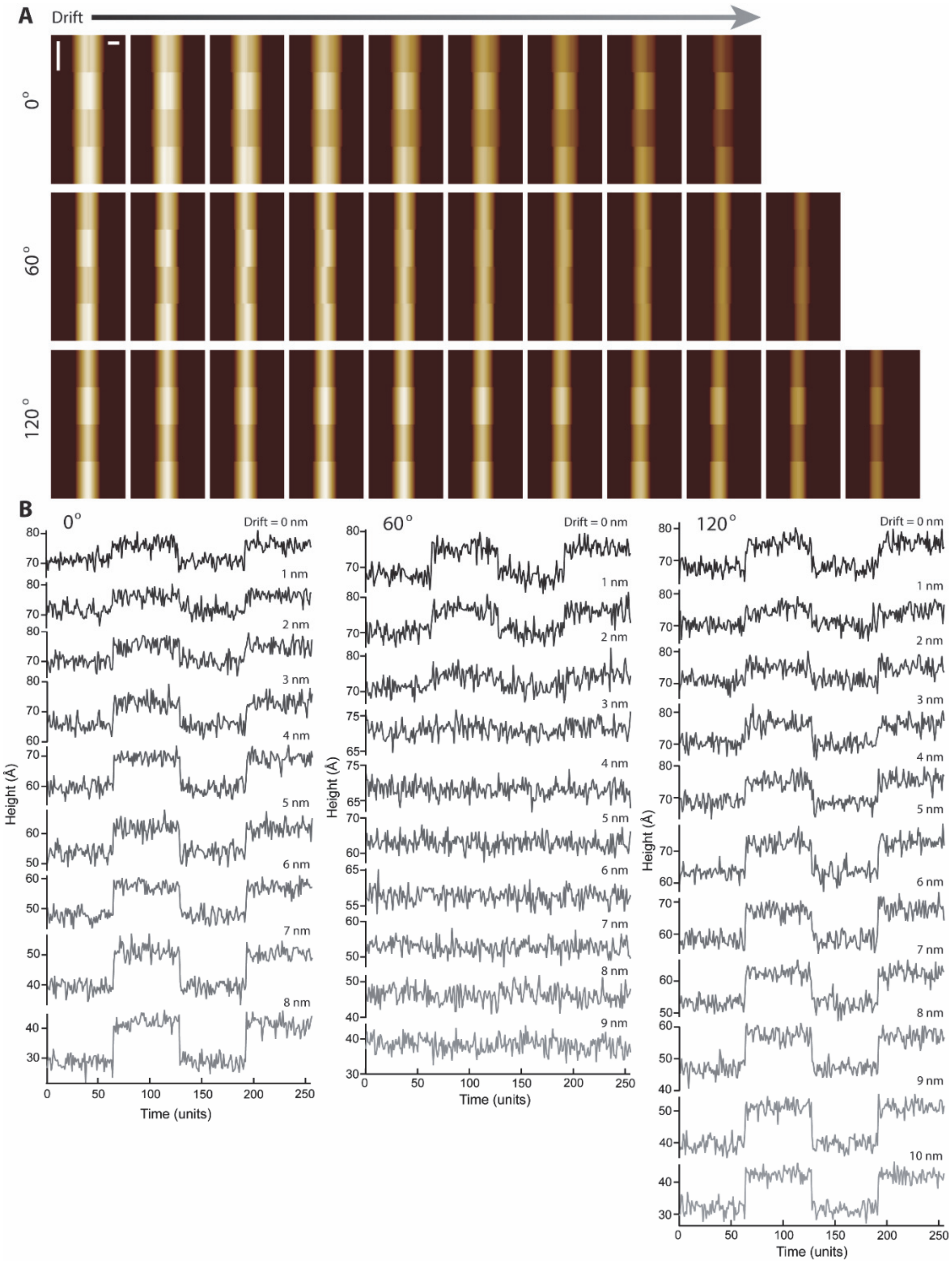

Figure S2. Kymograph simulation examples. (A) Simulated kymographs for 0°, 60°, and 120°. Due to the asymmetry of the protein, 0° scans could not drift as far as 120° scans before the tip loses contact with the protein. The vertical scale bar represents 50 time units and the horizontal scale bar is 30 Å. **(B)** Maximum heights are extracted from each kymograph and 2 Å Gaussian noise is added.

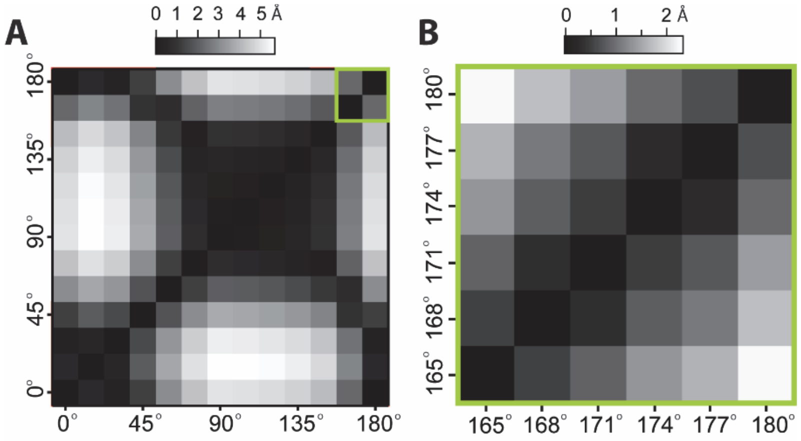

Figure S3. Resolved artifactual transitions. (A) A matrix of height differences, Δz, for rotations of the crystal structure of the IF state shows that few conformational rotations have a detectable change ($\Delta z > 2$ Å). A single instance of a 15° rotation having $\Delta z > 2$ Å is highlighted in green (165° to 180° and vice versa). **(B)** The boxed area from panel **A** is split into 3° rotations to make a matrix with higher resolution. This demonstrates that 15° is the lower angular displacement limit for erroneous transition detection.

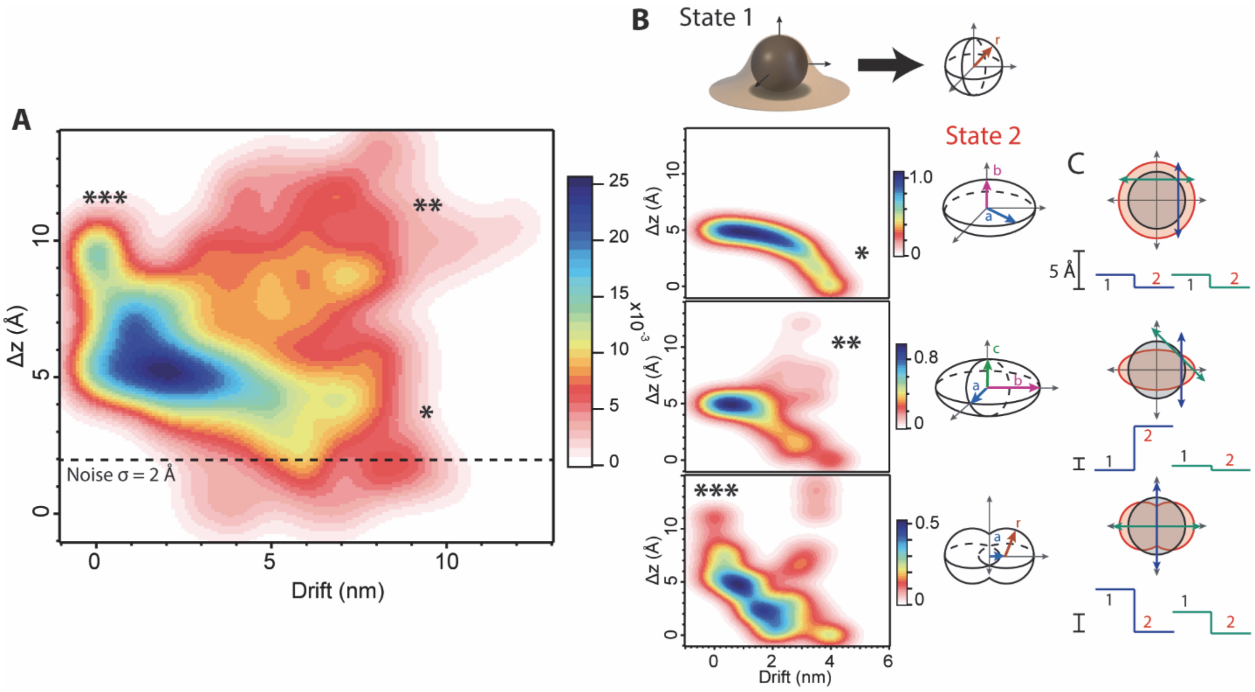

Figure S4. The effect of lateral drift on Δ*z*. (A) The height difference Δ*z* resulting from all scan angles is plotted in a heat map versus lateral drift. The vast majority of Δ*z*'s are above the 2 Å noise level (dashed line). Complex behavior can be isolated into three trends: (*) Δ*z* decreasing with drift, (**) Δ*z* increasing with drift, and (***) bimodal Δ*z* at the center of mass. **(B)** States are simplified into basic 2D geometries, a sphere for State 1, and three other shapes for State 2 listed in order from top to bottom: an oblate spheroid, an ellipsoid, and overlapping spheres. The resulting Δ*z* vs lateral drift heat maps correlate with behaviors observed in panel **A**. The false color scale bars are unitless, normalized probability density. **(C)** The two states are overlayed in a top view as a graphical representation of how different scan angles (blue and green) with similar drift can affect Δ*z*, giving rise to the behavior on corresponding heat maps. Transitions from state 1 to state 2 are shown as steps from the maximum *z*'s for each scan line. Note that despite changes in scale, the 5 Å value for the scale bar is preserved for each example.

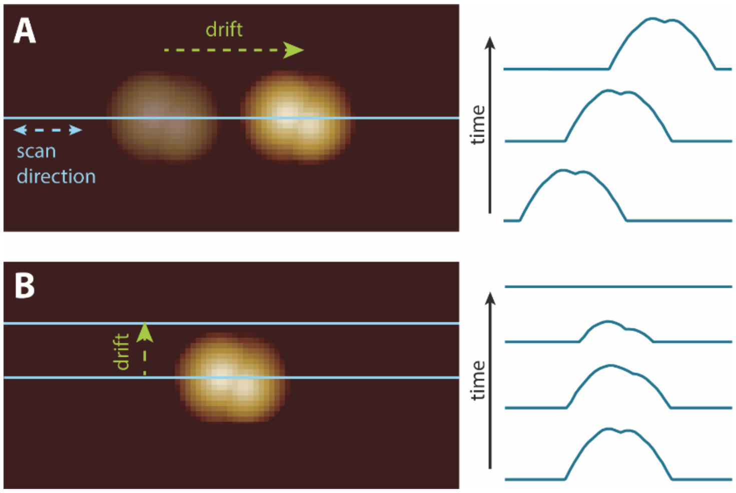

Figure S5. Drift directionality. (A) Drift along the kymograph axis (left panel) changes the feature location on the individual line scans (right panel), but will not reduce transition detection confidence as the tip still scans over the apex of the particle. **(B)** Drift perpendicular to the kymograph axis results in a change in height due to the displacement of the scan over the particle, therefore reducing confidence in transition detection.

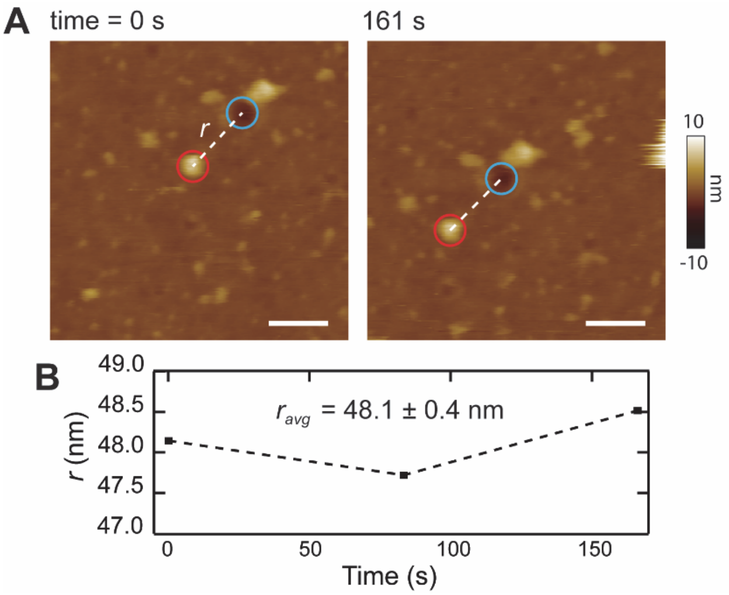

Figure S6. Lateral drift. (A) The first and last images in a stack of 5 images showing the drift of two features: a C-side Pgp particle (red) and a pore in the glass (blue). The displacement, *r*, is shown by the white dashed line, measured from the center of mass of each feature; scale bars = 40 nm. **(B)** For each time point, *r* is plotted, indicating that there is some variation in the displacement, but that the overall drift of the Pgp feature is registered with the glass pore.

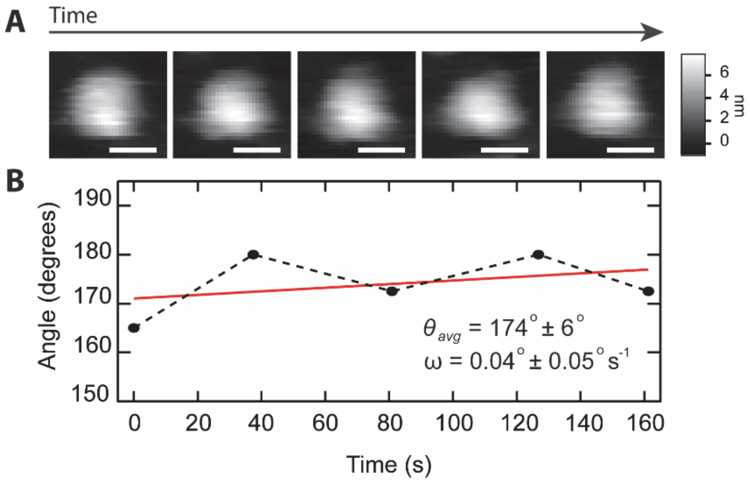

Figure S7. Rotational drift. (A) A single C-side feature is tracked in time; scale bars = 10 nm. **(B)** Quantitative agreement analysis [4] is performed with a simulated image (PDB: 7OTI [3]) to determine the angular orientation of the particle. The 0° angle is chosen to correspond with the orientation of the IF simulation in **Fig. 3A**. The angle is plotted versus time and a line fit (red) reveals slope, $\omega = \frac{d\theta}{dt}$. Kymographs were acquired over a temporal duration of <30 s. Hence, we expect an approximate 1° angular displacement due to rotational drift during this time (or equivalently, 6.4° over the 160 s imaging period shown).

References cited

1. Schwarz, G. Estimating the Dimension of a Model. *Ann. Stat.* **6**, 461–464 (1978).
2. Burnham, K. P. & Anderson, D. R. Multimodel Inference: Understanding AIC and BIC in Model Selection. *Sociol. Methods Res.* **33**, 261–304 (2004).
3. Barbieri, A. *et al.* Structure of ABCB1/P-Glycoprotein in the Presence of the CFTR Potentiator Ivacaftor. *Membranes* **11**, 923 (2021).
4. Chada, N. *et al.* Single-molecule observation of nucleotide induced conformational changes in basal SecA-ATP hydrolysis. *Sci. Adv.* **4**, eaat8797 (2018).
5. Chattrakun, K., Hoogerheide, D. P., Mao, C., Randall, L. L. & King, G. M. Protein Translocation Activity in Surface-Supported Lipid Bilayers. *Langmuir* **35**, 12246–12256 (2019).
